# The Three Gorges Dam: Does it accelerate or delay the progress towards eliminating transmission of schistosomiasis in China?

**DOI:** 10.1186/s40249-016-0156-3

**Published:** 2016-07-05

**Authors:** Yi-Biao Zhou, Song Liang, Yue Chen, Qing-Wu Jiang

**Affiliations:** Department of Epidemiology, School of Public Health, Fudan University, 138 Yi Xue Yuan Road, Shanghai, 200032 China; Key Laboratory of Public Health Safety, Ministry of Education, Fudan University, 138 Yi Xue Yuan Road, Shanghai, 200032 China; Center for Tropical Disease Research, Fudan University, 138 Yi Xue Yuan Road, Shanghai, 200032 China; Department of Environmental and Global Health, College of Public Health and Health Professions, University of Florida, Gainesville, FL USA; Emerging Pathogens Institute, University of Florida, Gainesville, FL USA; School of Epidemiology, Public Health and Preventive Medicine, Faculty of Medicine, University of Ottawa, 451 Smyth Road, Ottawa, Ontario Canada

**Keywords:** Three Gorges Dam, Schistosomiasis, *Schistosoma japonicum*, *Oncomelania hupensis hupensis*, Elimination, China

## Abstract

The Three Gorges Dam, located in the largest endemic area of schistosomiasis in China, is one of the world’s largest hydroelectric projects to date. Some large-scale hydro projects have resulted in schistosomiasis emergence or re-emergence. Therefore, the dam’s potential impact on the transmission of *Schistosoma japonicum* has raised concerns from medical researchers worldwide. A systematic literature review, coupled with an analysis of data on the water level and snail density in the Yangtze River was conducted to assess the impact of the dam on schistosomiasis transmission after more than 10 years of operation. The dam has significantly altered the water levels in the Yangtze River according to different seasons. These changes directly impact the ecology of the schistosome snail host. Due to the dam, there has been a reduction in the density of *Oncomelania* snails and/or changes in the distribution of snails. The prevalence of infection with *S. japonicum* has decreased in the downstream areas of the dam, including in the Dongting and Poyang Lakes. The prevalence of infection with *S. japonicum* in humans has decreased from 6.80 % in 2002 (before the dam began operating) to 0.50 % in 2012, and the number of people infected with *S. japonicum* have decreased from 94 208 in 2002 to 59 200 in 2011 in the Poyang Lake region. The presence of the dam does not seem to affect snail breeding or the prevalence of schistosomiasis in the Three Gorges Reservoir. Overall, the Three Gorges Dam has significantly contributed to changes in hydrology after more than 10 years of the dam operating. The changes caused by the dam, together with integrated control of schistosomiasis, might be accelerating the progress towards eliminating the transmission of *S. japonicum* in the middle and lower reaches of the Yangtze River. Despite the positive effect the dam is having in controlling *S. japonicum* transmission, continued surveillance is required to monitor the future ecological impacts of the dam over the long term.

**Electronic supplementary material:**

The online version of this article (doi:10.1186/s40249-016-0156-3) contains supplementary material, which is available to authorized users.

## Multilingual abstracts

Please see Additional file 1 for translations of the abstract into six official working languages of the United Nations.

## Background

**Fig. 1:**
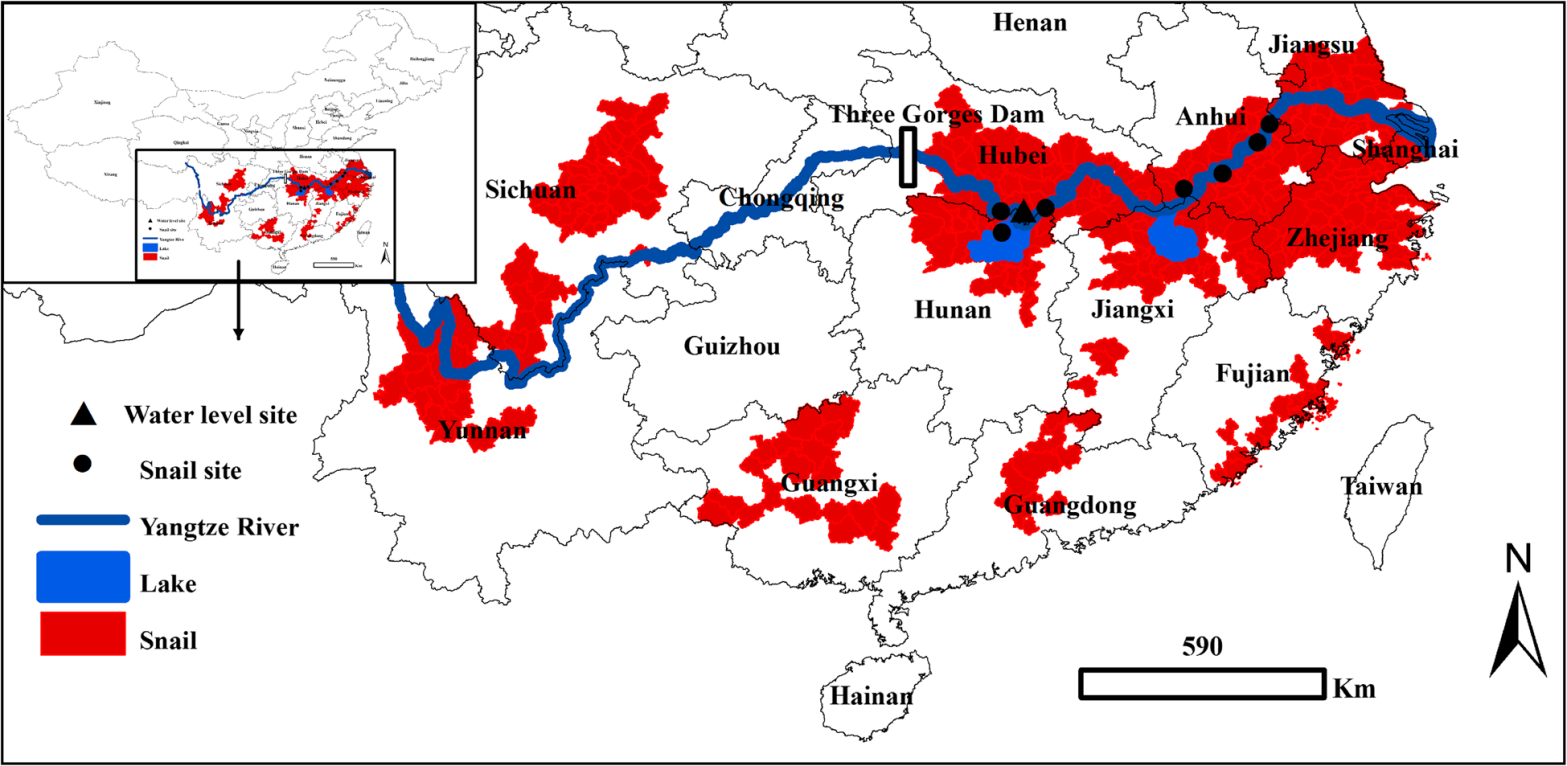
Location of the Three Gorges Dam, snail surveillance sites and hydrological stations on the Yangtze River, China

The Three Gorges Dam, the world’s largest dam, is located in the upper reaches of the Yangtze River, the longest river in Asia and the third longest in the world. The middle and lower reaches of the Yangtze River are the largest endemic area of schistosomiasis in China (see Fig. 1) [1]. The two largest lakes in China (Poyang Lake in Jiangxi Province and Dongting Lake in Hunan Province), which are located along the middle and lower reaches of the Yangtze River, constitute a shallow lake group unique in the world that extensively exchanges water with the river. The two lakes are endemic areas of schistosomiasis. The water level elevates substantially following the monsoon season. *Oncomelania hupensis hupensis* snails, the intermediate snail hosts, are distributed strictly in the Yangtze River basin due to its so-called ‘winter-land, summer-water’ ecohydrological condition that is favoured by the snails [2, 3].

The filling of the Three Gorges Reservoir began in June 2003, and its water level rose to 135 metres by the end of 2003, to 156 metres in 2006 and 172 metres in 2008. Since 2009, the water level has been maintained at 175 metres throughout November and December, and is lower in the other months.

It has previously been reported that some large-scale hydro projects (e.g., the Sudanese Gezira-Managil Dam, the Egyptian Aswan High Dam and the Ethiopian Melkasadi Dam) have resulted in schistosomiasis emergence or re-emergence [4–9]. Hence, the potential impact of the Three Gorges Dam on the transmission of *Schistosoma japonicum* in the Yangtze River basin has raised concerns from researchers worldwide [10–18]. Over the past decades, numerous studies have been carried out to forecast or assess the impact of the dam on the distribution of *O. h. hupensis* snails and the transmission of *S. japonicum* [19, 20].

Through a systematic review and an analysis of data on the water level and snail density, we assessed the dam’s impact on *S. japonicum* transmission after more than 10 years of operation. In this paper, we also discuss the potential implications for national strategies to control and eliminate schistosomiasis.

## Methods

### Search strategy

We conducted a systematic literature review by searching all relevant articles, published up to September 2015, which examined the impacts of the Three Gorges Dam on the transmission of schistosomiasis. Relevant studies were identified from the following electronic databases: PubMed, Science Citation Index Expanded™, China National Knowledge Infrastructure, Wanfang Data, China Science and Technology Journal Database and SINOMED. The following keywords and any combinations thereof were used: “Three Gorges Dam” in combination with “schistosomiasis”, “*Schistosoma*”, “snail” and “*Oncomelania hupensis*”. No language restrictions were applied.

### Article selection

Two reviewers independently checked the titles and abstracts of all identified articles for inclusion eligibility. We excluded the following: (1) review articles, (2) dissertations, (3) conference abstracts and presentations, and (4) non peer-reviewed reports. For eligible publications, full papers were retrieved and reviewed by the same two reviewers. They were then grouped into two categories: field simulation studies or prediction studies that were carried out before the dam started operating and therefore did not include data on the impact of the dam on schistosomiasis transmission in the study period; and observational or monitoring studies that were implemented after the dam started operating and were able to include data on the impact of the dam on schistosomiasis transmission in the study period.

### Data on changes in the water level and snail density

Daily data on the water level (above sea level, 8:00 AM) at the Chenglingji Hydrological Station (located at the junction of the Yangtze River and Dongting Lake) were collected from 1995 to 2013. In 2005, seven snail surveillance sites (villages) along the Yangtze River were set up to monitor the changes in the density of snails in the bottomland areas of the middle and lower reaches of the Yangtze River (see Fig. 1). In these snail surveillance sites, molluscicides for snail control were either not used or used very sparingly. These bottomland areas were surveyed using the traditional Chinese method of systematic sampling (20 m × 20 m) done annually in the spring [21]. Data on snail density (snail/0.11 m^2^) were collected from the seven snail surveillance sites from 2005 to 2013.

### Calculating the time of how long snail habitats are inundated with water

*O. h. hupensis* snails are distributed mainly in marshlands that are 25–28 metres in elevation in the Dongting Lake area close to the Chenglingji Hydrological Station [22]. Therefore, we calculated the time of 25 metre, 26 metre, 27 metre and 28 metre elevated marshes being inundated with water. If the water level in a day was higher than the elevation of the marsh, we regarded that marsh to be inundated with water. We estimated the link relative ratio of the days the marshes were inundated with water. The link relative ratio refers to the comparison between the days the marshes were inundated with water in a year and the days they were inundated with water in the previous year. For example, the days a marsh was inundated in 1996 are divided by the days it was inundated in 1995, thus achieving the link relative ratio of 1996.

## Results

### Included articles

**Fig. 2:**
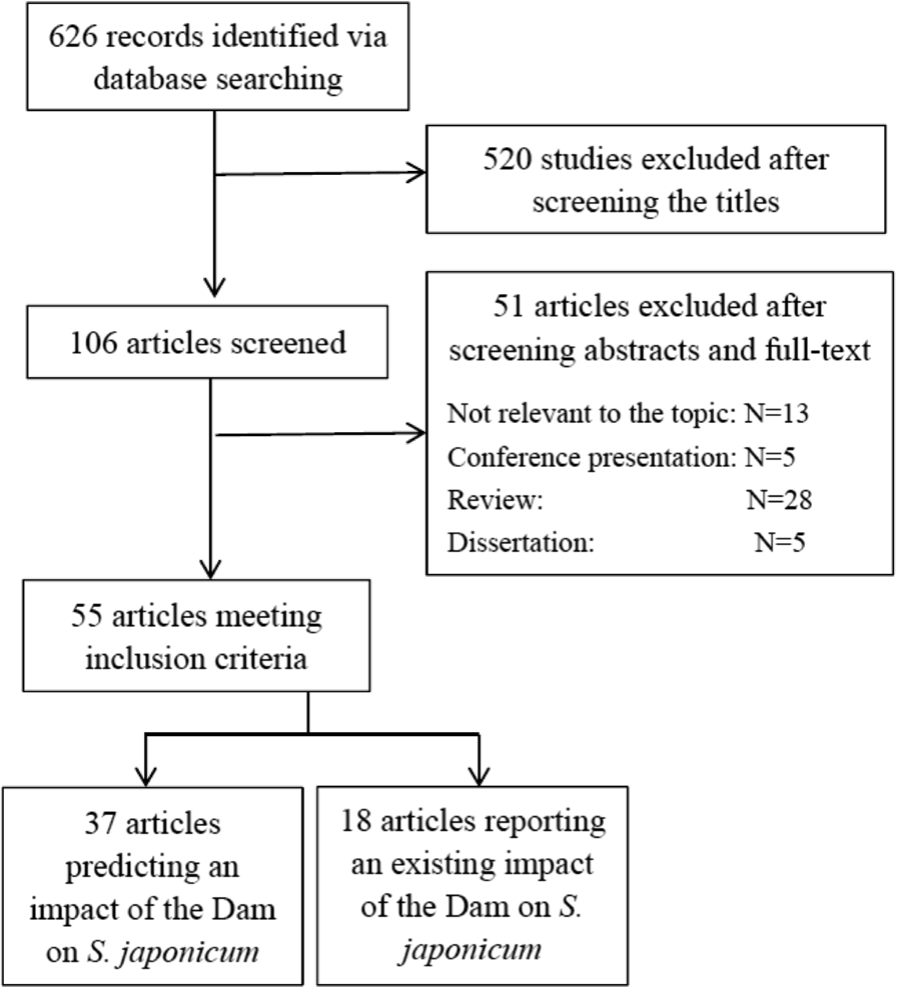
Decision tree showing inclusion or exclusion of articles found in the six electronic databases

A total of 626 articles were identified from six electronic databases (33 from PubMed, 37 from Science Citation Index Expanded™, 190 from Wanfang Data, 205 from China National Knowledge Infrastructure, 121 from China Science and Technology Journal Database and 40 from SINOMED). Due to duplication or irrelevance, 520 studies were excluded after the titles were screened. By screening the abstracts and full texts of the remaining 106 papers, it was determined that 51 articles would be excluded for they were not relevant to the topic, or they were review articles, dissertations or conference presentations, and 55 papers would be included in this study. The decision tree for the inclusion or exclusion of papers is illustrated in Fig. 2.

### Predicted impact of the dam on schistosomiasis transmission

A series of studies have been conducted predicting the potential impact of the Three Gorges Dam on the distribution of *Oncomelania* snails and transmission of *S. japonicum*. For the regions below the dam (i.e. along the middle and lower reaches of the Yangtze River), most of the studies consistently reported that the Three Gorges Dam would decrease the probability of flooding and cause changes in the water level in these areas of the Yangtze River; that is, upon completion of the dam, the water level would rise during the first part of the year and decrease during the last two or three months of the year (see Table 1). Most of the studies also consistently predicted that the decreased the probability of flooding due to the Three Gorges Dam would limit the dispersal of the snails and reduce the chance of human and livestock making contact with water that is infested with the cercariae of *S. japonicum*. However, most of the prediction studies we assessed reported that changes in the water level would increase the probability of human and livestock becoming infected with schistosomes, but that the epidemic status of schistosomiasis would be minimally affected in the Jiangxi segment of the Yangtze River (see Table 1). The predictions on the impact of the changes in the water level on *Oncomelania* snails were different in the different segments of the Yangtze River. For example, it has been predicted that some new habitats for *Oncomelania* snails would be created in the Hubei [23] and Anhui segments [24] of the river, but that the density of snails would decrease in the Jiangsu segment [25] after the completion of the dam (see Table 1). Some studies predicted that changes in the water level caused by the dam would not affect the reproduction or distribution of *Oncomelania* snails or increase the density of the snails [17, 20], but another study reported that changes in the water level would decrease the density of *Oncomelania* snails in the Jiangxi segment in autumn [13]. In addition, Cai et al. [26] reported in 2000 that the Three Gorges Dam would considerably reduce silt entering the Dongting Lake from the Yangtze River in the 50 years following the construction of the dam and that this would effectively limit the reproduction of snails.

**Table 1:** Predicted impact of the Three Gorges Dam on the distribution of *Oncomelania* snails and transmission of *S. japonicum*

Segment of Yangtze River	Water level rising		Water level decreasing		*Oncomelania* snails	*S. japonicum*	Refs.
	Height (m)	Months	Height (m)	Months			
Hubei (Jianghan Plain)	0.06–1.5	Jan – May	1.8–2.4	Nov, Dec	New habitats for *Oncomelania* snails would appear.	The probability of humans and livestock becoming infected would increase.	[17, 20, 23, 24]
Hunan (Dongting Lake)	0.06–1.5	Jan – May	1.6–2.6	Nov, Dec	The distribution of snails would not be significantly affected, however, the reproduction of snails would be effectively curbed.	The probability of humans and livestock becoming infected would increase and schistosomiasis epidemics would worsen.	[17, 20, 23, 25]
Jiangxi (Poyang Lake)	0.11–0.90	Jan – Mar	0.07–0.13	Dec	The reproduction and distribution of snails would be unaffected or the density of snails would increase, or the density of snails would decrease in the autumn.	The epidemiology of schistosomiasis would be very limitedly affected.	[17, 20, 23, 58, 59]
Anhui	0.14–0.76	Jan – Apr	0.06–1.26	Oct – Dec	The distribution of snails would not be significantly affected, however, some new habitats for snails would appear.	The probability of humans and livestock becoming infected would increase.	[17, 20, 24, 27, 60–62]
Jiangsu	0.15–0.40	Feb – Apr	0.32–0.75	Oct, Nov	The distribution of snails would be not affected and the density of snails would decrease.	The incidences of schistosome infections in people and livestock would increase.	[17, 20, 25, 27, 58]

Before the dam started operating, the Three Gorges Reservoir (see Fig. 1) was free for *Oncomelania* snails and *S. japonicum* for Yangtze water in the region was rapid following and collides with the cliffs, sandbars and precipices that flank the river [27]. Some studies reported that great environmental changes (e.g., sedimentation and formation of marshlands) resulting from the dam could form potential habitats for *Oncomelania* snails [27–29], whereas some simulated studies reported that *Oncomelania* snails could survive and breed in the Three Gorges Reservoir [30–34]. Some researchers reported that both *Oncomelania* snails and transmission sources of schistosomiasis might have been introduced into the Three Gorges region due to the dam [35–37]. However, other studies reported that the Three Gorges Reservoir would not be an ideal location for the reproduction of *Oncomelania* snails, as the dam’s ‘winter-water, summer-land’ operation cycle contradicts with the snails’ ‘winter-land, summer-water’ breeding cycle [38, 39].

### Dam’s impact on the water level

**Fig. 3:**
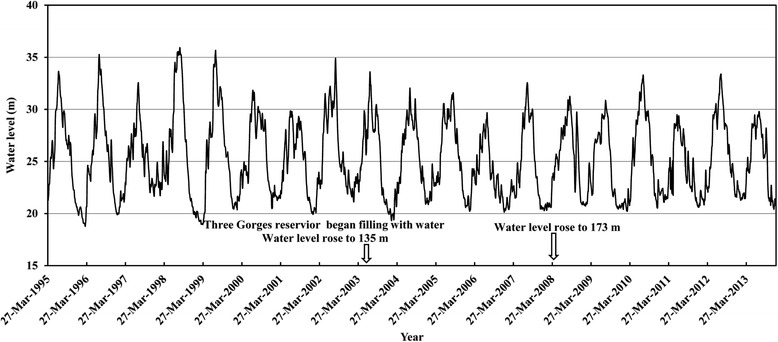
The daily water level above sea level (8:00 AM) at the Chenglingji Hydrological Station located at the junction of Yangtze River and Dongting Lake, from 1995 to 2013

After the Three Gorges Dam started operating, various studies have reported on the dam’s impact on the water level in the regions below the dam [40–46]. The results of these studies are consistent with or similar to each other: they report that the dam has effectively controlled floods and because of it, an early-flooded bottomland occurs in the spring and earlier water recessions occur in the autumn along the middle and lower reaches of the Yangtze River. For example, Li et al. [43] reported that the mean water level between January and March 2012 rose by 0.41 metres in the Jiangsu segment of the Yangtze River, and that from April to December 2012, it decreased by 0.32 metres, compared with the levels in 2002. These results are similar or consistent with the results from monitoring the Chenglingji Hydrological Station. Figure 3 shows that the peak water level decreased and the lowest water level rose in the Yangtze River from 1995 to 2013. During the first 10 years of the dam’s operation, the water levels were above 32.5 metres (warning line) in only three of the years and the water levels were above 33.0 metres (dangerous line) in only two of the years, compared with seven years and six years, respectively, during the nine years before the dam started operating (1995–2003).

**Fig. 4:**
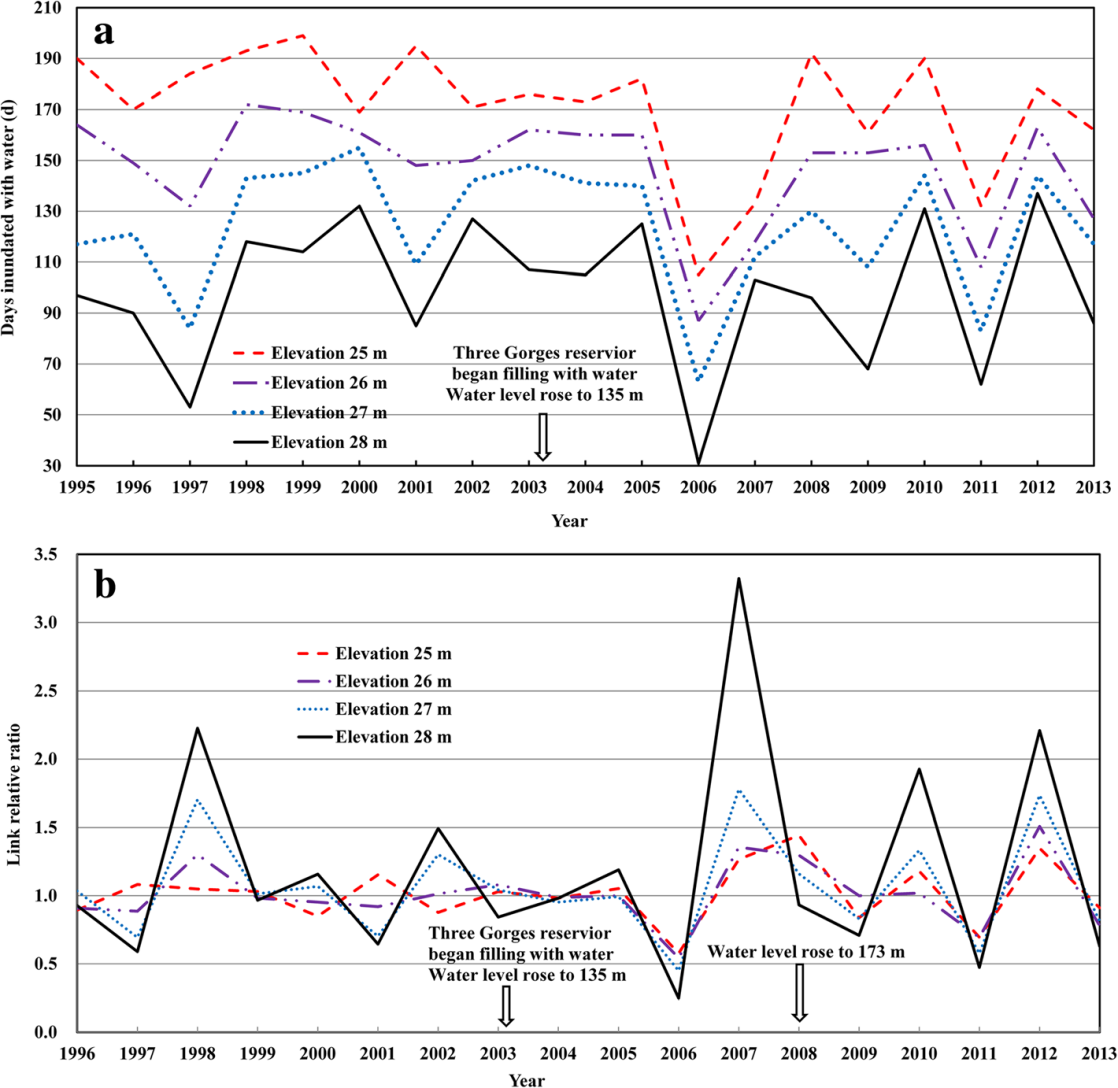
The number (**a**) and link relative ratio (**b**) of days that snail-harbouring marshland are inundated with water in the Dongting Lake region, from 1995 to 2013

The time that the snail-harbouring marshlands were inundated with water changed more frequently and in a wider range after the completion of the dam. Such changes in the time were marketed in 2006 that the time the snail habitats were covered with water was very short (see Fig. 4a) with a high link relative ratio (see Fig. 4b).

### Dam’s impact on schistosomiasis transmission

**Fig. 5:**
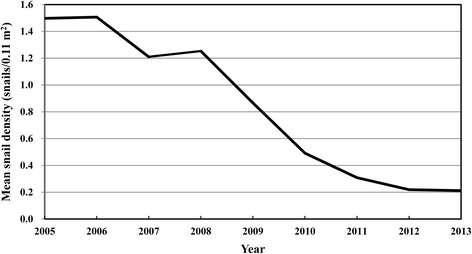
The density of *Oncomelania* snails in the bottomland areas of the middle and lower reaches of the Yangtze River, from 2005 to 2013

During the last several years, some studies have reported on the Three Gorges Dam’s impact on the distribution of *Oncomelania* snails and transmission of *S. japonicum* after the dam operating. Most of these reports revealed that the Three Gorges Dam has contributed to a reduction in the density of *Oncomelania* snails and/or changes in the distribution of *Oncomelania* snails in the downstream areas of the dam, including the Dongting and Poyang Lakes [19, 40, 42, 44–48]. This was consistent with the monitoring results. Figure 5 shows that the density of *Oncomelania* snails in the bottomland areas of the middle and lower reaches of the Yangtze River has decreased notably from 2005 to 2013. Some studies reported that both the prevalence of infection with *S. japonicum* and the number of individuals infected with *S. japonicum* have decreased significantly after more than 10 years of the dam’s operation [40, 41, 43, 45]. For example, Chen et al. [45] reported that the prevalence of infection with *S. japonicum* in humans decreased from 6.80 % in 2002 (before the dam started operating) to 0.50 % in 2012. They also reported that the number of individuals infected with *S. japonicum* decreased from 94,208 in 2002 to 59,200 in 2011, and that the number of acute *S. japonicum* cases decreased from 128 in 2002 to one in 2012 in the Poyang Lake region. Meanwhile, Zhang et al. [40] reported that the number of individuals infected with *S. japonicum* reduced from 54,304 in 2002 to 25,378 in 2012, and that the number of acute *S. japonicum* cases reduced from 251 in 2002 to two in 2012 in Anhui Province. However, other studies reported that the dam had virtually no immediate impact on the transmission of schistosomiasis [11, 49] and some other studies showed that the Three Gorges Dam does not definitely affect snail breeding and the prevalence of schistosomiasis in the Three Gorges Reservoir, as the dam operates in a so-called ‘winter-water, summer-land’ cycle. That is, the water levels rising in the winter and falling in summer in the reservoir contradict with the *Oncomelania* snails’ ‘winter-land, summer-water’ breeding stimulation. In particular, *Oncomelania* snails struggle to survive in high water levels in the winter, which is the situation due to the dam [38, 50, 51].

## Discussion

The construction of the Three Gorges Dam commenced in 1994 after decades of research and fierce debate. Benefits of the dam, such as flood control and power generation, are indisputable and the dam has lived up to these expectations. However, this study found that the dam’s impact on the transmission of *S. japonicum* is different to previous forecasts (see Table 1). Overall, this study found that the changes in hydrology caused by the Three Gorges Dam might be favourable for the control of *S. japonicum* transmission, based on the results from the observational and monitoring studies [19, 40–50].

There might be several reasons for this. Firstly, the dam has effectively controlled floods and deterred the dispersal of *Oncomelania* snails, and thus curtailed the infection to humans and animals [52, 53]. For example, some studies reported that the number of patients infected with *S. japonicum* reduced significantly, and especially that acute *S. japonicum* cases were rarely seen in the middle and lower reaches of Yangtze River after more than 10 years of the dam’s operation [40, 41, 45]. Secondly, during the first few months of the year, the presence of the dam leads to the production of an early flooded bottomland [43], and this not only can result in the death of adult *Oncomelania* snails or reduce their survival rate and oviposition, but is also not a favourable environment for embryo development of *Oncomelania* eggs [25]. Moreover, after the dam started operating, it has been observed that in the summer, many snail-harbouring marshlands are either not flooded or only flooded for a very short period of time. This is also not favourable for the development of juvenile snails, as *Oncomelania* snails have to live in water during the early stages of their development [54]. Some studies, together with our monitoring results, showed that the density of *Oncomelania* snails decreased significantly in many snail-harbouring marshes in the downstream areas of the dam [40, 45–48]. Thirdly, earlier water recessions in the autumn caused by the dam have advanced the exposure time of snail-harbouring marshes, thus they may be suitable for early planting of crops such as wheat and rapeseed. Currently, some of these marshlands have begun to be planted and this has resulted in a significant decrease in the density of *Oncomelania* snails [55]. Although some farmers spend a longer period of time working on the marshlands and therefore have a higher chance of coming into contact with cercariae-infected water, other people and livestock such as bovines, the primary infection source of *S. japonicum* transmission in China, are kept away from these marshlands, meaning that the probability of most humans and livestock becoming infected with *S. japonicum* decreases. In addition, many studies predicted that advanced water recessions in the autumn would lengthen the amount of time humans and livestock spend on the marshlands and increase the incidence of schistosome infections for both humans and livestock [17, 20]. However, an integrated control programme, aiming to reduce the roles of bovines and humans as infection sources, has been implemented in China since 2005 [56]. The control programme consists of agricultural mechanisation, fencing bovines, chemotherapy for humans and bovines, health education, provision of clean water and improved sanitation [57]. Hence, the programme has led to a reduction in the amount of time that humans and livestock spend on marshlands. Up until now, there have been no reports of increased incidence of schistosome infections due to earlier water recessions in the autumn. Fourthly, the dam operates in a so-called ‘winter-water, summer-land’ cycle, which contradicts with the *Oncomelania* snails’ ‘winter-land, summer-water’ breeding stimulation. In particular, *Oncomelania* snails struggle to survive in high water levels in the winter, which is the situation due to the dam [38, 50, 51]. Although both *Oncomelania* snails and human transmission sources for schistosomiasis may have been introduced from schistosomiasis endemic areas into the Three Gorges Dam region [20], no local sites of *Oncomelania* snails or locally infected people have been identified so far [19].

The changes in hydrology caused by the Three Gorges Dam may have an important implication for the Chinese national control strategies that aim to eliminate schistosomiasis. For example, tractors should plant crops such as wheat and rapeseed in the snail-harbouring marshlands that are suitable for early planting of crops due to early water recessions in the autumn. This can reduce the density of *Oncomelania* snails or even eliminate snails, as well as increase the income of farmers. Of course, farmers should be educated about how to avoid contact with cercariae-infected water when planting on these snail-harbouring marshlands.

Our study had some limitations. Although a large number of articles were found in the course of the systematic review, most of these were excluded due to duplication or irrelevance, and only 55 papers reporting the relationship between the Three Gorges Dam and the transmission of schistosomiasis were included in the study. Most of the included articles were simulation or forecast studies, or used only routine monitoring data to assess the dam’s impact on the transmission of schistosomiasis. Hence, it was difficult for us to systematically and quantitatively analyse the impacts of the Three Gorges Dam on the transmission of schistosomiasis. In view of this limitation, the relationship between the dam and the transmission of schistosomiasis outlined in this study should be interpreted with caution.

## Conclusion

There have been significant changes in hydrology since the Three Gorges Dam started operating in 2003. These changes, together with the integrated control programme currently implemented in China, might be accelerating the progress towards the elimination of *S. japonicum* transmission in the middle and lower reaches of the Yangtze River. Continued surveillance is needed to monitor the longer-term ecological impacts of the dam on the transmission of schistosomiasis.

## Additional file

Additional file 1: Multilingual abstracts in the six official working languages of the United Nations. (PDF 747 kb)

## References

[1] Ross AG, Sleigh AC, Li Y, Davis GM, Williams GM, Jiang Z, Feng Z, McManus DP (2001). Schistosomiasis in the People’s Republic of China: prospects and challenges for the 21st century. Clin Microbiol Rev.

[2] Zhou YB, Yang MX, Zhao GM, Wei JG, Jiang QW (2007). *Oncomelania hupensis* (Gastropoda: Rissooidea), intermediate host of *schistosoma japonicum* in China: Genetics and molecular phylogeny based amplified fragment length polymorphisms. Malacologia.

[3] Davis GM, Wilke T, Wu WP, Xu XJ (2006). Ecogenetics of shell sculpture in *Oncomelania* (Gastropoda) in canals of Hubei, China, and relevance for *Schistosome* transmission. Malacologia.

[4] Teklehaimanot A, Fletcher M (1990). A parasitological and malacological survey of schistosomiasis mansoni in the Beles Valley, northwestern Ethiopia. Trop Med Int Health.

[5] Fenwick A, Service MW (1989). Irrigation in the Sudan and schistosomiasis. Demography and Vector Born Diseases.

[6] Strickland G (1982). Providing health services on the Aswan High Dam. World Health Forum.

[7] Omer AHS (1975). Schistosomiasis in the Sudan: historical background and the present magnitude of the problem. Proceedings of the International Conference on Schistosomiasis.

[8] Amin MA, Worthington EB (1977). Problems and effects of schistosomiasis in irrigation schemes in the Sudan. Arid Land Irrigation in Developing Countries.

[9] Khalil BM (1949). The national campaign for the treatment and control of bilharziasis from the scientific and economic aspects. Royal Egyptian Medicine Association.

[10] Li YS, Raso G, Zhao ZY, He YK, Ellis MK, McManus DP (2007). Large water management projects and schistosomiasis control, Dongting Lake region, China. Emerg Infect Dis.

[11] Gray DJ, Thrift AP, Williams GM, Zheng F, Li YS, Guo J, Chen H, Wang T, Xu XJ, Zhu R, Zhu H, Cao CL, Lin DD, Zhao ZY, Li RS, Davis GM, McManus DP (2012). Five-year longitudinal assessment of the downstream impact on schistosomiasis transmission following closure of the Three Gorges Dam. PLoS Negl Trop Dis.

[12] Stone R (2011). Hydropower. The legacy of the Three Gorges Dam. Science.

[13] Seto EY, Wu W, Liu HY, Chen HG, Hubbard A, Holt A, Davis GM (2008). Impact of changing water levels and weather on *Oncomelania hupensis hupensis* populations, the snail host of *Schistosoma japonicum*, downstream of the Three Gorges Dam. Ecohealth.

[14] Engels D, Wang LY, Palmer KL (2005). Control of schistosomiasis in China. Acta Trop.

[15] Minter A (2005). Breeding snail fever. Three Gorges Dam boosts parasitic infections. Sci Am.

[16] McManus DP, Gray DJ, Li Y, Feng Z, Williams GM, Stewart D, Rey-Ladino J, Ross AG. Schistosomiasis in the People's Republic of China: the era of the Three Gorges Dam. Clin Microbiol Rev. 2010;23:442–66. 10.1128/CMR.00044-09 PMC2863366 20375361

[17] Zheng J, Gu XG, Xu YL, Ge JH, Yang XX, He CH, Tang C, Cai KP, Jiang QW, Liang YS, Wang TP, Xu XJ, Zhong JH, Yuan HC, Zhou XN (2002). Relationship between the transmission of schistosomiasis japonica and the construction of the Three Gorge Reservoir. Acta Trop.

[18] Maszle DR, Whitehead PG, Johnson RC, Spear RC (1998). Hydrological studies of schistosomiasis transport in Sichuan Province, China. Sci Total Environ.

[19] Wu JY, Zhou YB, Chen Y, Liang S, Li LH, Zheng SB, Zhu SP, Ren GH, Song XX, Jiang QW (2015). Three Gorges Dam: Impact of water level changes on the density of *Schistosome*-transmitting snail *Oncomelania hupensis* in Dongting Lake Area, China. PLoS Negl Trop Dis.

[20] Zhu HM, Xiang S, Yang K, Wu XH, Zhou XN (2008). Three Gorges Dam and its impact on the potential transmission of schistosomiasis in regions along the Yangtze River. Ecohealth.

[21] Zhou YB, Liang S, Chen GX, Rea C, He ZG, Zhang ZJ, Wei JG, Zhao GM, Jiang QW (2011). An integrated strategy for transmission control of *Schistosoma japonicum* in a marshland area of China: findings from a five-year longitudinal survey and mathematical modeling. Am J Trop Med Hyg.

[22] Luo ZH, Wei WY, Li ZJ, Ding L, Yuan LP, Xia M, Tang L, Ren GH, Wang JS, Wei GY (2012). Impact of environmental changes on *Oncomelania* snail distribution in Dongting Lake beach. Chin J Schisto Control.

[23] Xu XJ, Yang XX, Dai YH, Yu GY, Chen LY, Su ZM (1999). Impact of environmental change and schistosomiasis transmission in the middle reaches of the Yangtze River following the Three Gorges construction project. Southeast Asian J Trop Med Public Health.

[24] Wang TP, Ge JH, Zhang SQ (1998). Schistosomiasis transmission and ecological environmental changes in Anhui reach after construction of Three Gorges Reservoir. J Pract Parasi Dis.

[25] Liang YS, Huang YX, Song HT, Dai JR, Jiang YD, Man HC, Ji CS, Yang YY, Tian Q, Zhu YC (1999). Impact of variation in water level of the Yangtze River after the construction of the Three Gorges Dam on the transmission of schistosomiasis in Jiangsu, China. II. Observation of effect of advanced spring flooding on the population of snails and survey of frequency of contacting *schistosome*-infected water both for people and domestic animals in different season. Chin J Schisto Control.

[26] Cai KP, Zuo JZ, Ho HB, Zhuo SJ, Hu G (2000). Impact of changes in mud siltation of Dongting Lake on the endemic factors of schistosomiasis after building Three-Gorge Dam. Practical Prev Med.

[27] Zheng J, Gu XG, Xu CL, Wang TP, Xu XJ, He CH, Tang C, Cai KP, Jiang QW, Liang YS, Ge JH, Yang XX, Zheng QS, Yuan HC, Han JJ, Liang S, Wen S, Zuo JJ, Huang YX, Hu WZ, Wang YZ (2003). The relationship between the ecological changes in the construction of the Three Gorges Dam and the transmission of schistosomiasis. Bull Med Res.

[28] Lai J, Gu XG, Xu FS, Wen S, Liang S (2000). Study on the development of snail habitat in Three Gorges Reservoir area inundated temporarily by flood. J Pract Parasit Dis.

[29] He CH, Zhang AH, Guo SL, Pan HM, Lin CX, Wen YZ, Dong MJ, Han JJ (1998). Study on the effects of social and Economic changes on the spreading of schistosomiasis in Hubei area of the reservoir after construction of Three Gorge Dams. Med Soc.

[30] Xiao BZ, Liao WF, Wu CG, Ji HQ, Wu GH, Luo XJ, Wan SX, Lin XG (2008). The effect of ecological changes in the three Gorges reservoir areas on the prevalence of schistosomiasis. J Trop Med.

[31] Xiao BZ, Liao WF, Ji HQ, Wu CG, Wan SX, Lin XG (2004). Reproduction and Growth of *Oncomelania* snails under simulated biological environment in Three Gorges area. Chin J Schisto Control.

[32] Wei FH, Wang RB, Xu XJ, Xiao BZ, Wu XH, Liu JB, Cai SX, Fu Y, Xu J, Wu CG, Dai YH, Zhou XN, Zheng J (2007). Risk factors of schistosomiasis transmission after Three Gorges construction IPossibility of snails breeding with ecological changes in Three Gorges reservoir areas. Chin J Schisto Control.

[33] Zhou CY, Yang JS, Meng YP, Tang L (2004). Reproduction and Growth of *Oncomelania* snails under biological environment simulated as Three Gorges area. Chin J Schisto Control.

[34] Wang RB, Xu XJ, Xiao BZ, Wei FH, Wu XH, Zhou XN, Zheng J (2003). Study on the possibility of snail breeding after the ecological changes of the Three Gorges Reservior areas. J Trop Med.

[35] Wu CG, Xiao BZ, Liao WF, Yan W (2005). Analysis of the epidemiological factors of schistosomiasis in the Three Gorges Reservoir areas. J Trop Med.

[36] Wei FH, Wang RB, Xu XJ, Liu JB, Fu Y, Zhang J (2004). Investigation on import way of schistosomiasis and *Oncomelania* snails in Three Gorges Reservoir areas. Chin J Schisto Control.

[37] Xu FS, Wen S, Qian XH, Mao Y, Zhong B, Lei J, Gu XG, Wang MP (1999). The impact of social factors on schistosomiasis prevalence at the Three Gorges Reservoir areas. J Pract Parasit Dis.

[38] Xuan Y, Wang XL, Qu XH, Chen ZJ, Gao YH, Yang XM, Zhang Y, Pan HX (2012). Study on influence of Three Gorges project construction on *Oncomelania* growth condition in Chongqing section. Chin J Schisto Control.

[39] Xiao RW, Ye JF, Tao LF (1998). Study on *Oncomelania* snail breeding and spreading in the Three Gorges Reservoir region. Collected Works of Ecology and Environment Impact on Three Gorges Project.

[40] Zhang SQ, Wang TP, He JC, Li HZ, Tian XG, Gao F (2015). Impact on prevalence of schistosomiasis after runs of Three Gorges Reservoir Project in the section of Anhui province. Chin J Prev Med.

[41] Chen YY, Cai SX, Xiao Y, Shan XW, Zhang J, Liu JB (2014). Impact of implementation of Three Gorges Project on schistosomiasis endemic situation in Hubei Province. Chin J Schisto Control.

[42] Li ZJ, Chen HG, Zeng XJ, Liu YM, Chen YY, Dai KJ, Lan WM, Xie SY (2014). Studies on changes of vegetation and *Oncomelania hupensis* snails in Poyang Lake after impoundment of Three Gorges Project. Chin J Schisto Control.

[43] Li W, Hang DR, You BR, Chen XJ, Chen XJ, Yang K, Liang YS (2013). Effect of environmental change in marshland after implementation of Three Gorges Reservoir Project on schistosomiasis in Jiangsu Province. Chin J Schisto Control.

[44] Zhu R, Zhou YB, Zhang LJ, He ZY, Xu XL, Guo JG, Zhao GM (2013). The impact of water level changes on the *Oncomelania* snail habitats in Poyang Lake regions before and after the impoundment of Three Gorges reservoir. Chin J Prev Med.

[45] Chen HG, Zeng XJ, Lin DD, Lv SB, Gu XN, Hang CQ, Li ZJ (2013). The changes of hydrological regime in Poyang Lake after runs of Three Gorges Project and its impact on prevalence of schistosomiasis in the lake region. Chin J Schisto Control.

[46] Guo FY, Zhao ZY, Ren MY, Liu GC, Hu BJ, Xia M (2012). Impacts of marshland changes on snail distribution in Dongting Lake after the construction of the Three Gorges Dam. J Trop Dis Parasit..

[47] Zhu CF, Zeng QF, Li YY, Lu SB, Huang WJ (2015). Variation of *oncomelnia* and schistosomiasis in midstream area of Yangtze River after operation of Three Gorges Reservoir. Yangtze River.

[48] Zeng QF, Zhu CF, Li YY (2015). Influence of Three Gorges reservoir operation on *oncomelnia* and schistosomiasis in Dongting Lake region. Yangtze River.

[49] Zhu R, Gray DJ, Thrift AP, Williams GM, Zhang Y, Qiu DC, Zheng F, Li YS, Guo J, Zhu HQ, Wu WP, Li RS, McManus DP (2011). A 5-year longitudinal study of schistosomiasis transmission in Shian village, the Anning River Valley, Sichuan Province, the Peoples’ Republic of China. Parasit Vectors.

[50] Xuan Y, Chen ZJ, Qu XH, Gao YH, Yang XM (2012). The Study of Three Gorges Project on *Oncomelania hupensis* of Chongqing Area. Chin J Zool.

[51] Zhou YB, Zhuang JL, Yang MX, Zhang ZJ, Wei JG, Peng WX, Zhao GM, Zhang SM, Jiang QW (2010). Effects of low temperature on the *schistosome*-transmitting snail *Oncomelania hupensis* and the implications of global climate change. Molluscan Res.

[52] Wu XH, Zhang SQ, Xu XJ, Huang YX, Steinmann P, Utzinger J, Wang TP, Xu J, Zheng J, Zhou XN (2008). Effect of floods on the transmission of schistosomiasis in the Yangtze River valley, People’s Republic of China. Parasitol Int.

[53] Zhou YB, Liu QL, Zhao ZY, Chen Y (1998). Survey report on the snail diffusion of 5 villages in Dongting Lake area after flood. Chin J Schisto Control.

[54] Zheng YJ, Zhong JH, Chen XL, Lin DD, Zhao GM, Zhang SJ, Jiang QW (2002). Influence of drowning on survival of Oncomelania. Chin J Schisto Control.

[55] Tao HY, Xia A, Zhao YM, Jiang J (2012). Effect and cost-benefit of *Oncomelania* snail control by plowing and planting in Jiaobei Beach of Zhenjiang City. Chin J Schisto Control.

[56] Zhou YB, Liang S, Jiang QW (2012). Factors impacting on progress towards elimination of transmission of schistosomiasis japonica in China. Parasit Vectors.

[57] Wang LD, Guo JG, Wu XH, Chen HG, Wang TP, Zhu SP, Zhang ZH, Steinmann P, Yang GJ, Wang SP, Wu ZD, Wang LY, Hao Y, Bergquist R, Utzinger J, Zhou XN (2009). China’s new strategy to block *Schistosoma japonicum* transmission: experiences and impact beyond schistosomiasis. Trop Med Int Health.

[58] Wang RB, Zheng J (2003). Three Gorges Dam project and the transmission of schistosomiasis in China. Chin J Schisto Control.

[59] Zhang SJ, Yu ZH, Wu ZD (1995). The impact of water level change in Jiangxi Province caused by construction of the Three Gorges Dam on the transmission of schistosomiasis in Poyang Lake. J Jiangxi Prev Med.

[60] Wu CG, Zhou XN, Xiao BZ (2005). A comprehensive summary of the relationship between the ecological changes in the construction of the Three Gorges Dam and the transmission of *S. japonicum*. Parasit Dis Foreign Med Sciences.

[61] Zhang SQ, Wang TP, Ge JH, He JC, Tao CG, Zhang GH, Lu DB, Wu WD (2000). The impact of the Three Gorges Dam construction on the exploitation and utilization of marshland and the prevalence of schistosomiasis in Anhui Province. Chin J Parasit Dis Control.

[62] Zhang SQ, Ge JH, Zhang GH (1998). The impact of water level change in Anhui Province caused by Three Gorges Dam construction on behaviours of people and cattle and schistosomiasis prevalence. Chin J Parasit Dis Control.

