# Supplementary material for: The Three Gorges Dam: Does it accelerate or delay the progress towards eliminating transmission of schistosomiasis in China?
Source: Infect Dis Poverty. 2016 Jul 5;5:63. doi: 10.1186/s40249-016-0156-3 (PMC4932735; doi:10.1186/s40249-016-0156-3)
Supplement: Additional file 1: — Multilingual abstracts in the six official working languages of the United Nations. (PDF 747 kb) [file 40249_2016_156_MOESM1_ESM.pdf]

Translation of the abstract into the six official working languages of the United Nations

سُدّ الممرات الثلاثة: هل يُساهم في تسريع القضاء على انتشار داء البلهارسيات في الصين أو الحدّ من ذلك؟

يي- بياو تشو، سونغ ليانغ، يو تشين وتشينغ - ووجيانغ

مُلخّص

تقديم عام: يقع سُدّ الممرات الثلاثة في أكبر منطقة ينتشر فيها داء البلهارسيات في الصين ويُعتبر من أكبر مشاريع توليد الطاقة الكهرومائية في العالم إلى الآن. تسبّبت بعض المشاريع المائية في ظهور داء البلهارسيات أو عودة ظهوره. لذلك، فإن تأثير السُدّ المحتمل في انتشار البلهارسية اليابانية (*Schistosoma japonicum*) قد زاد من مخاوف الباحثين في المجال الطبي في جميع أنحاء العالم.

التقنيات المستخدمة: أُجريت مراجعة منهجية للوثائق إضافة إلى تحليل البيانات المتعلقة بمستوى المياه و عدد الحلزون في نهر يانغتسي بهدف تقييم تأثير السُدّ على انتشار داء البلهارسيات بعد أكثر من 10 سنوات من تشغيل السدّ.

النتائج: أحدث السُدّ تغييرا كبيرا فيما يتعلق بمستويات المياه في نهر اليانتغسي خلال مواسم مختلفة وقد أثرت هذه التغييرات مباشرة على البيئة المُحتوية الحلزون الحامل للبلهارسيات. كما أنه أدّى إلى انخفاض عدد الحلزون (*Oncomelania*) و/أو تغييرات على مستوى انتشاره. انخفاض معدل انتشار عدوى البلهارسية اليابانية (*S.japonicum*) في مناطق المصب للسد بما في ذلك في بحيرات دونجتنج وبويانغ وقد انخفض معدل العدوى بالبلهارسية اليابانية عند البشر من 6.80% سنة 2002 (قبل تشغيل السُدّ) إلى 0.50% سنة 2012، إذ انخفض عدد المصابين بالبلهارسية اليابانية من 94208 سنة 2002 إلى 59200 سنة 2011 في منطقة بحيرة بويانغ. يبدو أنّ وجود السُدّ ليس له تأثير على تكاثر الحلزون أو على انخفاض انتشار البلهارسيات في خزان الممرّات الثلاثة. وعمومًا، ساهم سدّ الممرات الثلاثة بشكل كبيرفي تغيير الهيدرولوجيا بعد أكثر من 10 سنوات من تشغيل السد.

الخاتمة: مكّنت التغييرات الناجمة عن السُدّ، إضافة إلى مراقبة انتشار داء البلهارسيات من تسريع القضاء على انتشار البلهارسية اليابانية (*S.japonicum*) في الضفاف الوسطى والسفلى لنهر يانغتسي. وبالرغم، من التأثير الإيجابي للسدّ في التحكّم في انتشار البلهارسية اليابانية فالأمر يتطلب مراقبة مستمرة لرصد الآثار البيئية المستقبلية للسد على المدى الطويل.

Translated from English version into Arabic by Zeineb Trabelsi, through

TRANSLATORS WITHOUT BORDERS

三峡大坝是加速还是延缓中国消除血吸虫病传播的进程？

周艺彪，梁松，陈跃，姜庆五

摘要：

三峡大坝是世界上迄今为止最大的水利工程之一，它坐落在中国最大的血吸虫病流行区。一些大的水利工程已经导致了血吸虫病流行与再现。因此三峡大坝对血吸虫传播的可能影响吸引了全球医学研究者的广泛关注。在三峡大坝运行10多年后，采用系统文献综述和分析长江水位及其洲滩钉螺密度数据来评估大坝对血吸虫病传播的影响。三峡大坝已明显改变了长江的水位。这种水位的变化对血吸虫中间宿主钉螺的生态产生了直接的影响，导致钉螺密度的下降或改变了钉螺的分布。在大坝的下游地区，包括洞庭湖和鄱阳湖地区，血吸虫病感染率明显下降。在鄱阳湖地区，人群血吸虫病感染率从2002年的6.80%下降到2012年的0.50%。血吸虫病病人数从2002年的94,208人降低到2011年的59,200人。大坝似乎没有导致三峡库区钉螺的孳生和血吸虫病的流行。总之，在三峡大坝运行10多年后，长江的水文发生明显的改变。在长江中下游地区，三峡大坝所致的变化与目前实施的血吸虫病综合防治措施可能加速了血吸虫传播的消除进程。尽管三峡大坝目前对血吸虫病的控制是有利的，但仍有必要继续监测大坝对生态的长期影响。

Translated from English version into Chinese by Zhou Yi-Biao

Le barrage des Trois Gorges : accélérateur ou frein à l'élimination de la transmission de la schistosomiase en Chine ?

Yi-Biao Zhou, Song Liang, Yue Chen and Qing-Wu Jiang

Résumé

Contexte : Le barrage des Trois Gorges est l'un des plus grands projets hydroélectriques au monde. Il est par ailleurs situé dans la plus vaste zone de schistosomiase endémique de Chine, or certains projets hydrologiques à grande échelle ont favorisé l'émergence ou la réémergence de la maladie. Les chercheurs médicaux du monde entier se sont donc inquiétés de l'impact potentiel du barrage sur la transmission de *Schistosoma japonicum*.

Méthodes : Une revue systématique de la littérature, couplée à l'analyse des données relatives au niveau d'eau et à la densité des mollusques hôtes dans le fleuve Yangzi, a été menée afin d'évaluer l'effet du barrage sur la transmission de la schistosomiase, plus de 10 ans après sa mise en eau.

Résultats : Le barrage a considérablement modifié le régime saisonnier du Yangzi et ces changements ont eu un impact direct sur l'écologie de l'escargot *Oncomelania*, hôte du schistosome. La densité de ce mollusque a diminué et/ou sa distribution a changé depuis la construction du barrage. La prévalence de l'infestation par *S. japonicum* a diminué en aval du barrage, notamment dans les lacs Dongting et Poyang. La prévalence de *S. japonicum* chez l'homme a diminué de 6,80 % en 2002 (avant la mise en eau du barrage) à 0,50 % en 2012, et le nombre de personnes infestées par *S. japonicum* de 94 208 en 2002 à 59 200 en 2011 dans la région du lac Poyang. La présence du barrage ne semble pas influer sur la reproduction des mollusques ni sur la prévalence de la schistosomiase dans le bassin des Trois Gorges. Globalement, le barrage des Trois Gorges a significativement contribué aux changements hydrologiques, plus de dix ans après sa mise en service.

Conclusion : Les changements causés par le barrage pourraient s'ajouter aux mesures intégrées de lutte contre la schistosomiase pour accélérer l'élimination de la transmission de *S. japonicum* sur le cours moyen et inférieur du fleuve Yangzi. Malgré l'effet positif du barrage sur la lutte contre *S. japonicum*, la surveillance doit être maintenue afin de déterminer l'impact écologique futur de l'ouvrage à long terme.

Translated from English version into French by Suzanne Assenat, through

TRANSLATORS WITHOUT BORDERS

Три ущелья-Плотина: это ускорит ли прогресс в направлении ликвидации или отсрочить передачу шистосомоза в Китае?

Чжоу-Бяо, Сун Лян, Чэнь и Цин-Ву Цзян Юэ

Аннотация

Цель: Три ущелья-Плотина, расположенный в по величине в Китае эндемичных местности шистосомоза является одним из крупнейших гидроэнергетических проектов в мире на сегодняшний день. Некоторые крупные гидропроекты привели к возникновению шистосомоза или повторному возникновению. Таким образом, потенциальное воздействие плотины на передачу Schistosoma japonicum вызывает озабоченность медицинских исследователей во всем мире.

Методы: систематический обзор литературы, в сочетании с анализом данных об уровне воды в реке Янцзы и улитка

плотности было проведено с целью оценки влияния плотины на передачу шистосоматоз после более чем 10 лет эксплуатации.
Результаты: Изменены уровни воды в плотине значительно разные времена года, в зависимости от реки Янцзы. Эти изменения оказывают непосредственное влияние на экологию хозяина улитки шистосом. Из-за плотины, наблюдается снижение плотности улитки Oncomelania и / или изменения в распределении улиток. Распространенность инфекции с S. Japonicum в нижерасположенных участках крыши, включая озер Дунтинху и Поянху уменьшилось. С распространением инфекции у человека Japonicum C. снизилась с 6,80% в 2002 году (до того, как плотина начала функционировать) до 0,50% в 2012 году, а число людей, инфицированных *S. japonicum* уменьшилось с 59 200 до 94 208 в 2002 году в 2011 году в озере Поянху этого региона. Наличие плотины, кажется, не влияет на распространенность шистосомоза или разведение улитки в Трех ущелий водохранилище. В целом, изменения в плотине Три ущелья-плотина, внесла значительный вклад в гидрологии после более чем 10 лет эксплуатации плотины.
Заключение: Изменения, вызванные плотиной, вместе со встроенным модулем управления шистосомоза, может ускорить прогресс в направлении устранения передачи*S. japonicum* в среднем и нижнем течении реки Янцзы. Плотина, несмотря на положительный эффект от управления передачей*S. japonicum*, постоянный надзор обязан следить за будущих экологических последствий плотины в долгосрочном периоде.

Translated from English version into Russian by Turdimurot Rakhmonov, through

La presa de las Tres Gargantas: ¿acelera o demora el proceso de eliminación de la transmisión de la esquistosomiasis en China?

Yi-Biao Zhou, Song Liang, Yue Chen y Qing-Wu Jiang

Resumen

Antecedentes: La presa de las Tres Gargantas, ubicada en el área endémica más extensa de esquistosomiasis en China, es uno de los proyectos hidroeléctricos más grandes del mundo hasta la fecha. Algunos proyectos hidroeléctricos a gran escala han provocado el surgimiento o resurgimiento de la esquistosomiasis. Por lo tanto, el impacto potencial de la presa sobre la transmisión del parásito *Schistosoma japonicum* dio origen a una preocupación por parte de investigadores médicos de todo el mundo.
Métodos: Se llevó a cabo una revisión global de la bibliografía, junto con el análisis de los datos sobre el nivel del agua y la densidad de la población de caracoles en el río Yangtze para evaluar el impacto de la presa sobre la transmisión de la esquistosomiasis después de más de 10 años de actividad.
Resultados: La presa ha modificado ampliamente los niveles de agua en el río Yangtze según las diferentes estaciones. Estos cambios influyen directamente en la ecología del huésped (caracol) del esquistosoma. Debido a la presa, ha habido una reducción en la densidad de la población de caracoles *Oncomelania* y/o cambios en la distribución de caracoles. La prevalencia de la infección por *S. japonicum* ha disminuido en las áreas corriente abajo de la presa, incluidos los lagos Dongting y Poyang. La prevalencia de la infección por *S. japonicum* en seres humanos ha disminuido de 6,80 % en 2002 (antes de que comenzara la actividad de la presa) a 0,50 % en 2012, y la cantidad de personas infectadas por *S. japonicum* disminuyó de 94 208 en 2002 a 59 200 en 2011 en la región del lago Poyang. La presencia de la presa no parece afectar la alimentación de los caracoles ni la prevalencia de la esquistosomiasis en el reservorio de las Tres Gargantas. En general, la presa de las Tres Gargantas ha contribuido de manera significativa con los cambios en la hidrología después de más de 10 años de actividad de la presa.
Conclusión: Es posible que los cambios originados por la presa, junto con el control integrado de la esquistosomiasis, estén

acelerando el proceso de eliminación de la transmisión del parásito *S. japonicum* en el radio medio y bajo del río Yangtze. A pesar del efecto positivo que tiene la presa para controlar la transmisión del parásito *S. japonicum*, se requiere una vigilancia continua para supervisar el impacto ecológico futuro de la presa en el largo plazo.

Translated from English version into Spanish by Mónica Belén Colacilli, through

TRANSLATORS
WITHOUT BORDERS
